# Supplementary material for: Building Bricks of Integrated Care Pathway for Autism Spectrum Disorder: A Systematic Review
Source: Int J Mol Sci. 2023 Mar 26;24(7):6222. doi: 10.3390/ijms24076222 (PMC10094376; doi:10.3390/ijms24076222)
Supplement: Supplementary file 1 [file ijms-24-06222-s001.zip › ijms-2216456-supplementary.pdf]

Documents provided by the ASDEU members

Country	*Document provided by the ASDEU members*
Bulgaria	Measures formally approved by the Ministry of Health (2013) to improve the care of people with ASD and their carers call for a better collaboration between public bodies from health, education, and social welfare sectors, in addition to closer cooperation with patient and parent associations. The document “Мерки за подобряване на грижите в помощ на лица с аутизъм и техните семейства” is available upon request.
Castile and Leon	Plan de acción para las personas con trastorno del espectro autismo en castilla y león. The document based on the Spanish Strategy on ASD and the "Strategic Plan for Equal Opportunities for Persons with Disabilities" of Castilla y León, in accordance with the singularities and realities existing in this Autonomous Community (https://serviciossociales.jcyl.es/web/es/dependencia-discapacidad/plan-estrategico-igualdad-oportunidades.html)
Castile la Mancha	Estrategia en trastorno del espectro del autismo para castilla-la mancha (2016). The document based on the Spanish Strategy on ASD and whose content has been adapted to the context of Castilla-La Mancha (https://www.autismocastillalamancha.org/wp-content/uploads/2020/08/Estrategia-Regional.pdf)
Catalonia	Pla director de salut mental i addiccions. Pla d’atenció integral a les persones amb trastorn de l’espectre autista. Barcelona: Direcció General de Regulació, Planificació i Recursos Sanitaris, Generalitat de Catalunya 2012 (https://www.autisme.com/autisme/documents/PlaAtencioTEA.pdf)
France	No specific laws for care for autistic people are available at national level. There is a law related to Disability in general (Loi du 11 février 2005 - https://handicap.gouv.fr/la-loi-du-11-fevrier-2005-pour-legalite-des-droits-et-des-chances?amp). Since 2005, the Haute Autorité de Santé HAS has been working on helping health professionals in their daily practices with autism: from identification and diagnosis to the implementation of educational and therapeutic interventions from childhood to adulthood (https://www.has-sante.fr/jcms/c_2829216/fr/autisme-travaux-de-la-has)<br>Additional information:<br>https://www.gouvernement.fr/argumentaire/strategie-nationale-pour-l-autisme-2018-2022-changeons-la-donne
Iceland	Best Practice Guidelines for Diagnosing Autism Spectrum Disorder in Children and Youth (https://www.greining.is/is/fraedsla-og-namskeid/hagnytt-efni-1; https://www.greining.is/static/files/kliniskar-leidbeiningar-um-greiningu-einhverfu-hja-bornum-og-ungmennum-desenmber-2021.pdf) . The organizational structure of services is reported in the document “Service for adults with autism in Iceland: summary of organizational structure and documentation” (2016)
Ireland	Development process for Autism Innovation Strategy. Minister of State with Special Responsibility for Disability (2021) (https://www.gov.ie/en/press-release/cc4b3-minister-rabbitte-announces-development-process-for-autism-innovation-strategy/)<br>Other additional documents:<br>https://www.oireachtas.ie/en/debates/debate/dail/2021-04-29/8/<br>https://www.oireachtas.ie/en/bills/bill/2017/61/

Italy	National law -No. 134 of August 18, 2015- titled Provisions in matter of diagnosis, care, and habilitation of people with ASD and assistance to their families. In 2016, the Italian Ministry of Health established the "Fund for the care of individuals with ASD" to ensure the full implementation of the national law. In 2018, occurred the updating of the guidelines for promoting and improving the quality and appropriateness of care interventions in ASD, Agreement for the plan between the Government, the Regions, the Autonomous Provinces and the Local Authorities. http://www.regioni.it/news/2018/05/14/conferenza-unificata-del-10-05-2018-intesa-sul-documento-recante-aggiornamento-delle-linee-di-indirizzo-per-la-promozione-ed-il-miglioramento-della-qualita-e-dellappropriatezza-degli-interve-561197/
Poland	Several law acts that provide a framework for medical care for autistic people. The list is available upon request.
Portugal	No specific laws for care for autistic people are available at national level. Guidelines for education and employability are general for persons with Disabilities. The documents are available upon request
Romania	Law no. 151/2010 for specialized integrated services - health, education, social services - dedicated to persons with ASD (https://legislatie.just.ro/Public/DetaliiDocumentAfis/120487). Additional documents are available upon request (protocol for diagnosis of ASD in children and adults and the protocol of Romanian Ministry of Health for family doctors).
Spain	Estrategia Española en Trastornos del Espectro del Autismo (2015) approved by the Ministry of Health, Social Services and Equality. The Spanish Strategy on Autism Spectrum Disorder (ASD) is the reference framework in the definition of state, autonomic and local policies and actions (https://www.mdsocialesa2030.gob.es/derechos-sociales/discapacidad/docs/Estrategia_Espanola_en_TEA.pdf)
UK	National strategy for autistic children, young people and adults: 2021 to 2026. The government's national strategy for improving the lives of autistic people, their families, and carers in England, and implementation plan for 2021 to 2022 https://www.gov.uk/government/publications/national-strategy-for-autistic-children-young-people-and-adults-2021-to-2026<br>Other guidelines and legislation:<br>• https://www.legislation.gov.uk/ukpga/2009/15/contents<br>• https://www.autism.org.uk/what-we-do/campaign/not-enough/about-the-autism-act<br>• https://www.nice.org.uk/guidance/conditions-and-diseases/mental-health-and-behavioural-conditions/autism<br>Additional strategies specific for personalization of care in social care and health:<br>• https://www.scie.org.uk/personalisation/specific-groups/autistic-spectrum-conditions<br>• https://www.cqc.org.uk/guidance-providers/autistic-people-learning-disability/right-support-right-care-right-culture<br>• https://www.nice.org.uk/guidance/qs51/chapter/quality-statement-3-personalised-plan

Risk of bias

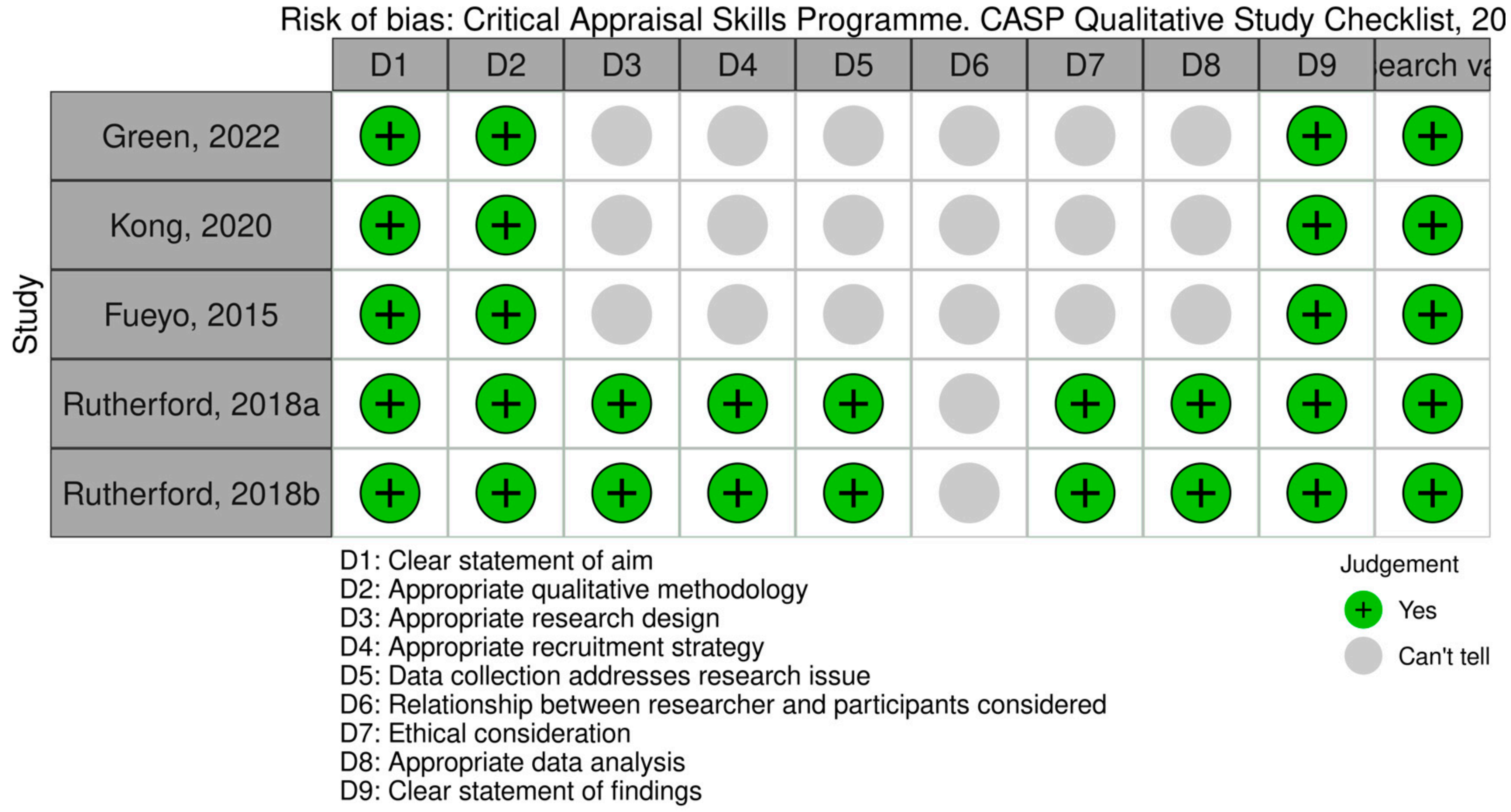
Risk of bias: Critical Appraisal Skills Programme. CASP Qualitative Study Checklist, 20
D1
D2
D3
D4
D5
D6
D7
D8
D9
earch va
Study
Green, 2022
Kong, 2020
Fueyo, 2015
Rutherford, 2018a
Rutherford, 2018b
D1: Clear statement of aim
D2: Appropriate qualitative methodology
D3: Appropriate research design
D4: Appropriate recruitment strategy
D5: Data collection addresses research issue
D6: Relationship between researcher and participants considered
D7: Ethical consideration
D8: Appropriate data analysis
D9: Clear statement of findings
Judgement
Yes
Can't tell
